# Supplementary material for: Horizontal transfer of a 180-kbp genomic fraction among the largest viral genomes
Source: Appl Environ Microbiol. 2026 Jun 18;92(7):e00105-26. doi: 10.1128/aem.00105-26 (PMC13390436; doi:10.1128/aem.00105-26)
Supplement: Supplemental material — Table S2; Fig. S1 to S6. [file aem.00105-26-s0001.pdf]

Supporting information

Horizontal transfer of a 180-kbp genomic fraction among the largest viral genomes

Hiroyuki Hikida*‡, Ruixuan Zhang, Jingjie Chen, Yusuke Okazaki, Hiroyuki Ogata

Chemical Life Science, Institute for Chemical Research, Kyoto University, Uji, Kyoto 611-0011, Japan

*Corresponding author: hikida.h@jihs.go.jp

Table S1 (Separate Excel file)

Table S2

Table S3 (Separate Excel file)

Figure S1–S6

‡Present address: Department of Virology I, National Institute of Infectious Diseases, Japan Institute for Health Security, 1-23-1, Toyama, Shinjuku, Tokyo 162-8640, Japan; Research Center for Biosafety, Laboratory Animal and Pathogen Bank, National Institute of Infectious Diseases, Japan Institute for Health Security, 1-23-1, Toyama, Shinjuku, Tokyo 162-8640, Japan

Table S2. List of viral genomes used in this study

Virus name	Accesssion	Abbreviation	Clade
Pandoravirus neocaledonia	MG011690.1	PanV_neo	Clade B
Pandoravirus aubagnensis	MZ420563.1	PanV_aub	Clade B
Pandoravirus japonicus	LC625835.1**	PanV_jap	Clade A-I
Pandoravirus pampulha strain Biwa	LC870878	PanV_biw	Clade A-I
Pandoravirus lena*	OQ411594.1	PanV_len	Clade A-II
Pandoravirus lena*	OQ411595.1	PanV_len	Clade A-II
Pandoravirus lena*	OQ411596.1	PanV_len	Clade A-II
Pandoravirus lena*	OQ411597.1	PanV_len	Clade A-II
Pandoravirus lena*	OQ411598.1	PanV_len	Clade A-II
Pandoravirus lena*	OQ411599.1	PanV_len	Clade A-II
Pandoravirus pampulha strain 8.5	LT972219.1	PanV_pam	Clade A-I
Pandoravirus salinus	KC977571.1	PanV_sal	Clade A-II
Pandoravirus mammoth*	OQ411600.1	PanV_mam	Clade B
Pandoravirus mammoth*	OQ411601.1	PanV_mam	Clade B
Pandoravirus dulcis	KC977570.1	PanV_dul	Clade A-I
Pandoravirus talik	OQ413801.1	PanV_tal	Clade A-II
Pandoravirus massiliensis isolate BZ81 c	MZ384240.1	PanV_mas	Clade B
Pandoravirus celtis	MK174290.1	PanV_cel	Clade A-II
Pandoravirus kuranda isolate Kuranda	ON887157.1	PanV_kur	Clade B
Pandoravirus inopinatum isolate KlaHel	KP136319.1	PanV_ino	Clade A-II
Pandoravirus belohorizontensis	MZ420562.1	PanV_bel	Clade A-I
Pandoravirus quercus	MG011689.1	PanV_que	Clade A-II
Pandoravirus macleodensis	MG011691.1	PanV_mac	Clade B

*Contigs are registered separately.

**re-assembled in this study

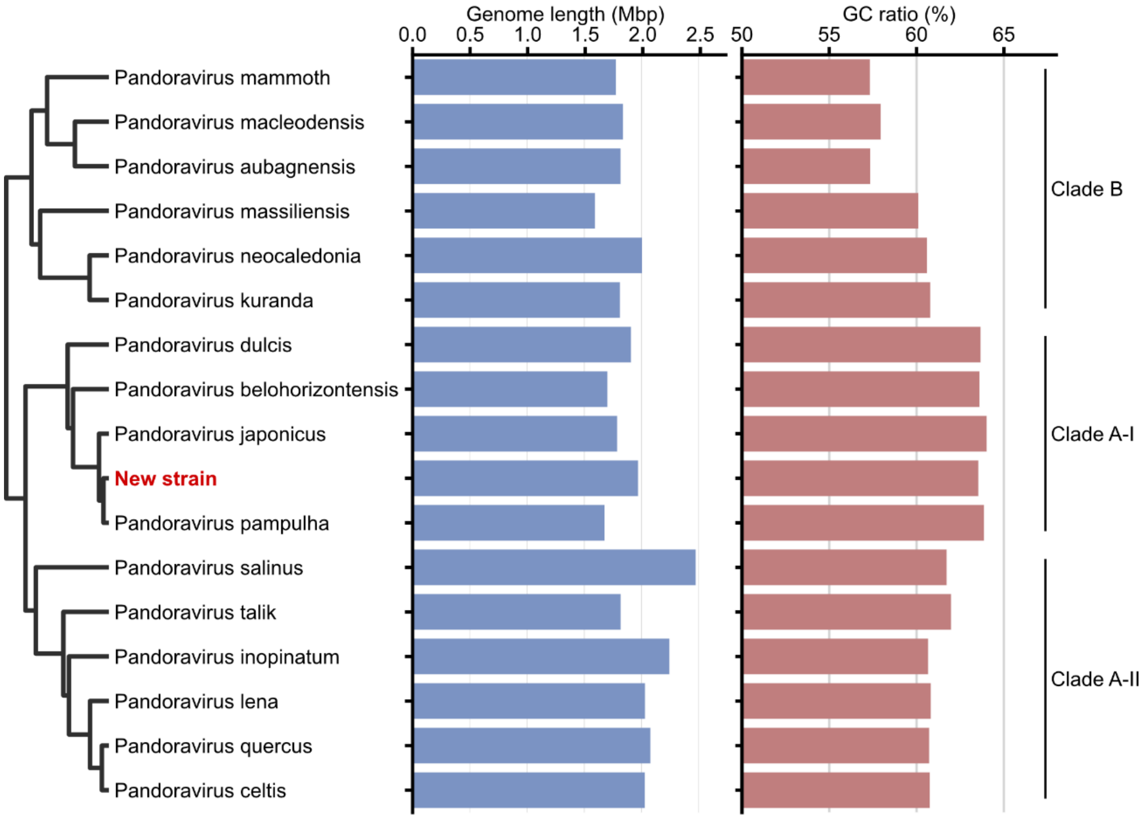

Figure S1. Genomic properties of pandoraviruses.

Length and GC ratio of pandoravirus genomes. The left dendrogram represents clustering based on the average nucleotide identity shown in Figure 1C.

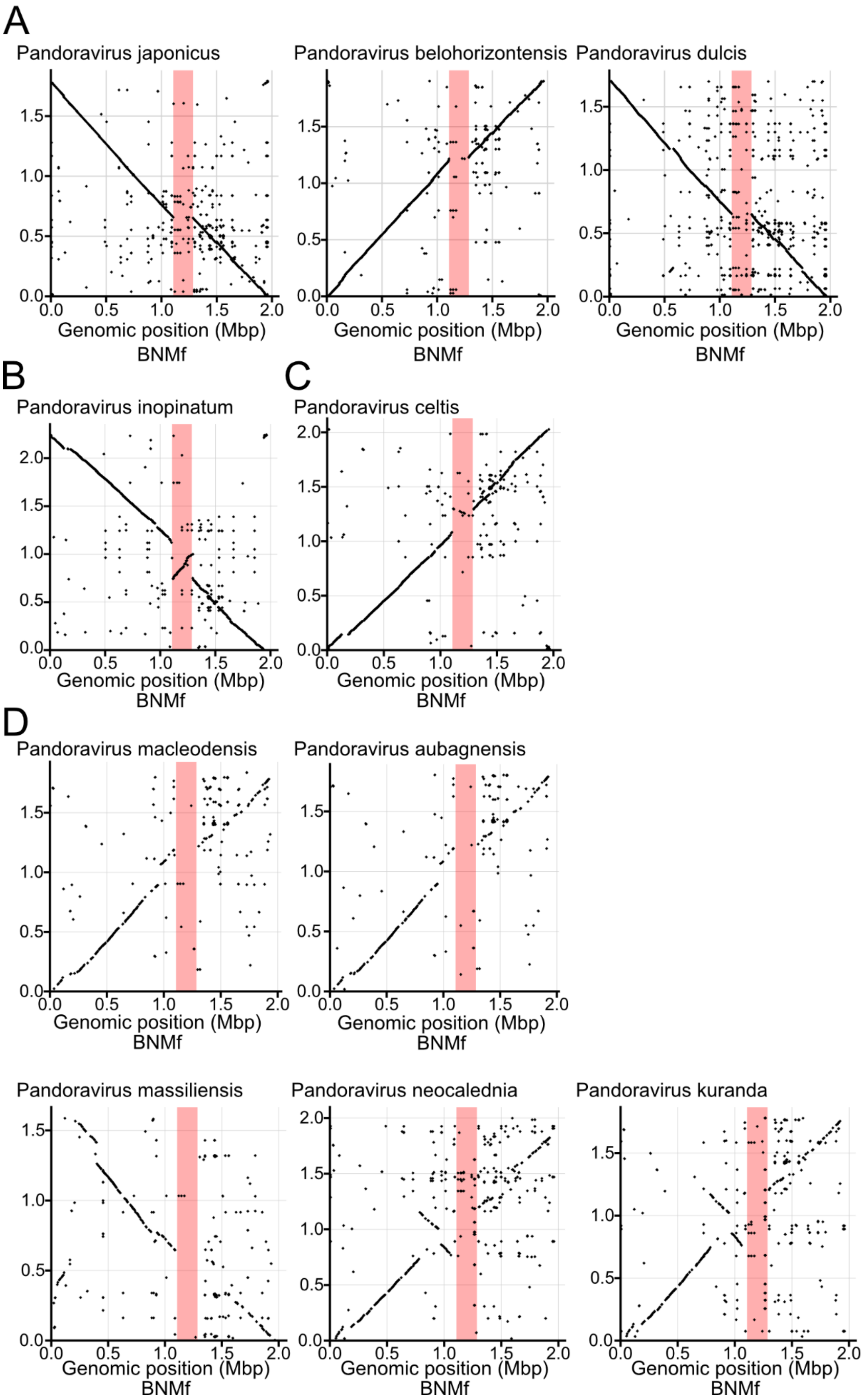
A
Pandoravirus japonicus
Pandoravirus belohorizontensis
Pandoravirus dulcis
B
Pandoravirus inopinatum
C
Pandoravirus celtis
D
Pandoravirus macleodensis
Pandoravirus aubagnensis
Pandoravirus massiliensis
Pandoravirus neocalednia
Pandoravirus kuranda
Genomic position (Mbp)
BNMf
0.0
0.5
1.0
1.5
2.0

Figure S2. Genomic comparison between PanV-biw and the other pandoraviruses.

Genomic comparison between PanV-biw and (A) pandoraviruses belonging to Clade A-I, (B, C) Clade A-II, and (D) Clade B. The X- and Y-axes represent the genomic position in PanV-biw and other viruses, respectively. The viruses are designated at the top left. Red boxes indicate the 180-kbp region missing in other Clade A-I viruses.

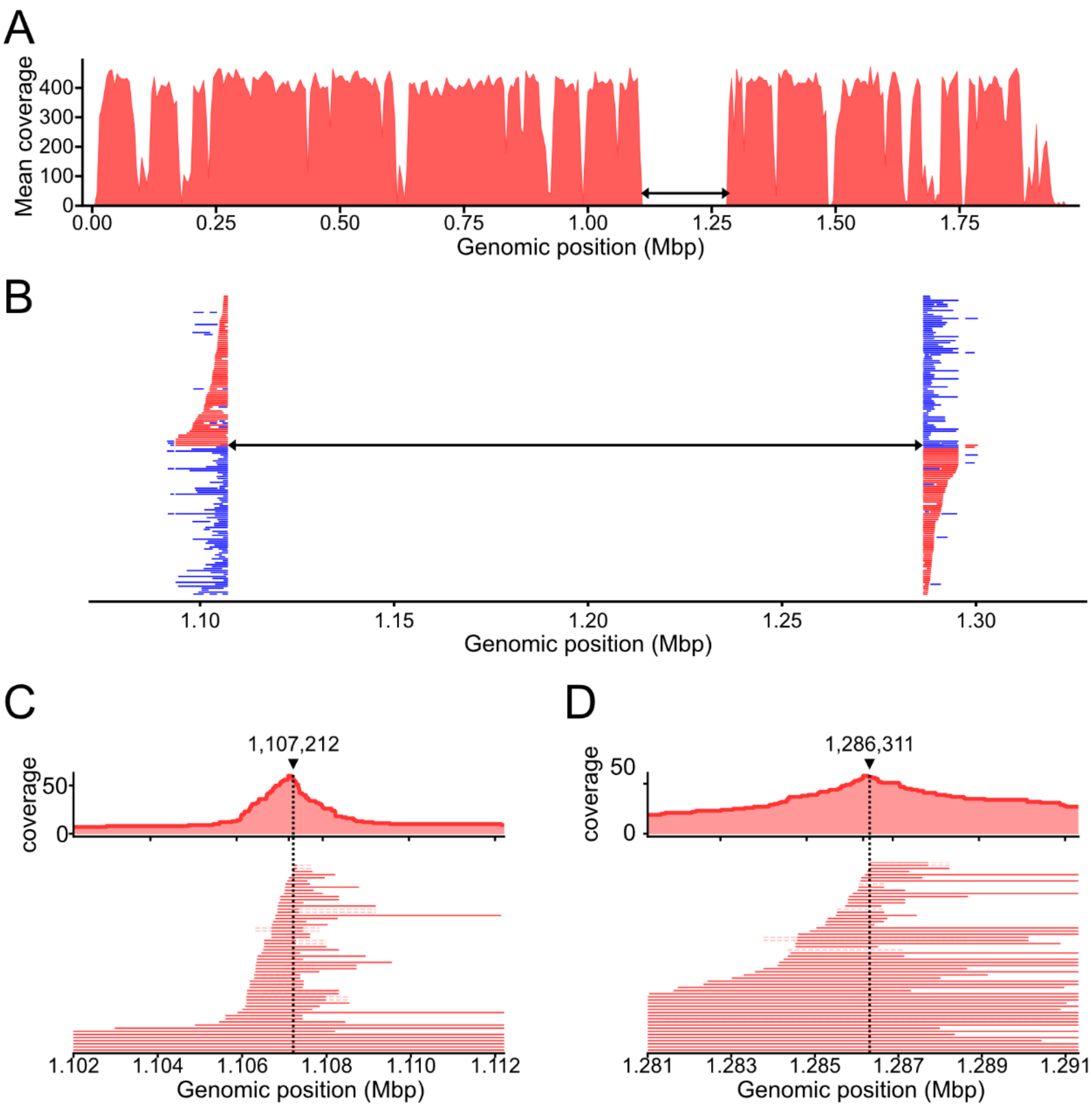

Figure S3. Read-mapping results for PanV-jap and PanV-biw.

(A) Raw long reads from PanV-jap were mapped to the PanV-biw genome. (B) Enlarged image around the 180-kbp region in (A). Each row represents a long read that bridged the 5′ and 3′ flanking regions of the 180-kbp region. The primary and secondary alignments of each read are shown in red and blue, respectively. (A, B) The black arrow indicates the 180-kbp region missing in PanV-jap. (C, D) Raw long reads of PanV-biw were mapped to the PanV-biw genome. Only reads that bridge the 180-kbp region and its flanking regions were shown. (C) 5′ and (D) 3′ boundary between the 180-kbp and flanking regions.

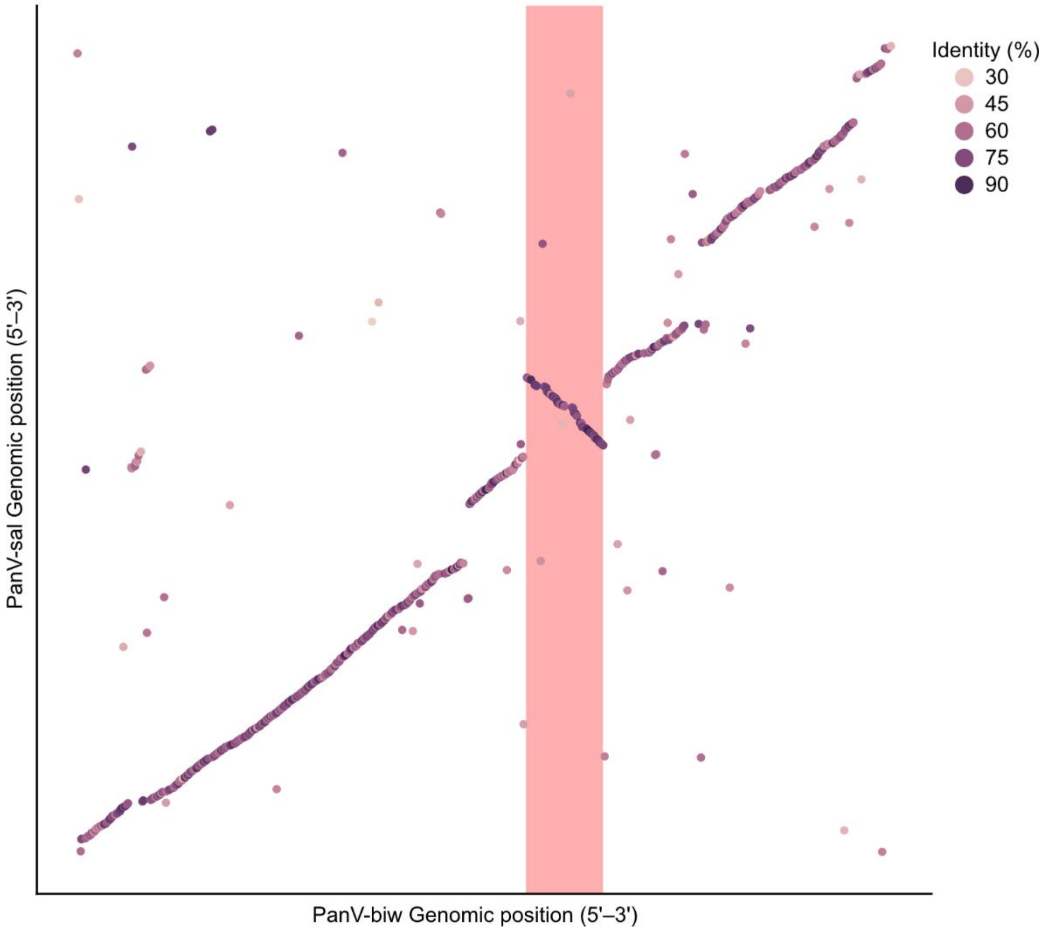

Figure S4. Consistent synteny between PanV-biw and PanV-sal.

Protein-level synteny between PanV-biw and PanV-sal was visualized from the data used in Figure 3A. The X- and Y-axes indicate the genomic positions of PanV-biw and PanV-sal, respectively. The colors indicate the identity of the proteins. Red boxes indicate the 180-kbp region in PanV-biw.

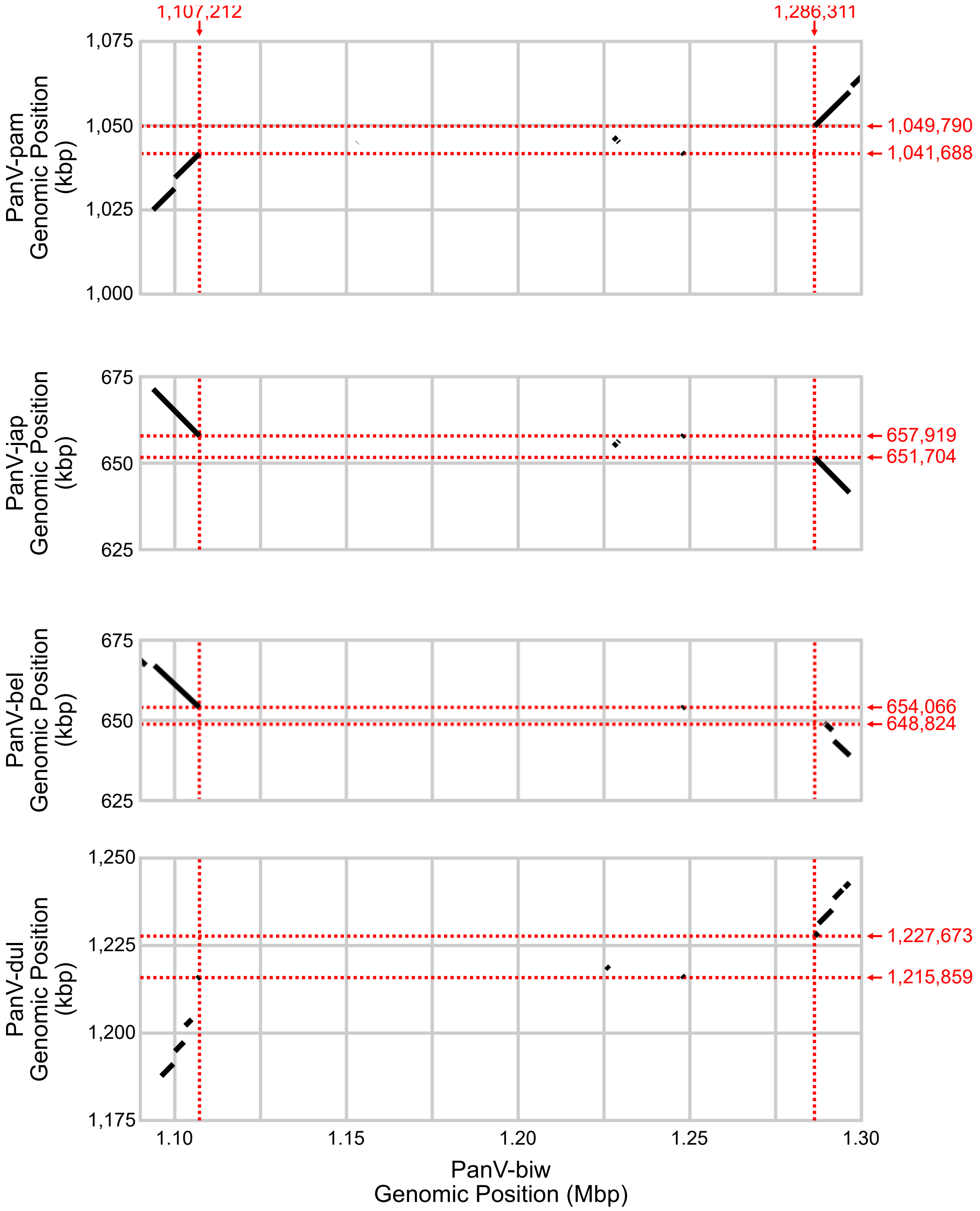

Figure S5. Enlarged dot plots around the regions corresponding to the 180-kbp region in PanV-biw

Genomic comparison between PanV-biw and pandoraviruses belonging to Clade A-I. Flanking regions of the 180-kbp region were enlarged. The X- and Y-axes represent genomic positions in PanV-biw and Clade A-I pandoraviruses, respectively. The viruses are designated on the left. The vertical and horizontal red dotted lines indicate the manually determined boundaries of the 180-kbp region and the corresponding regions of each Clade A-I pandoraviruses.

Pandoravirus pampulha Biwa

TGTCGACATAGATGG | ACTCGATCGCGAGCA---GCCGTTGCCCACCGT | TACCCGCCACATCCT

Pandoravirus salinus

ATAGTCGTTCTCGTA | CCACTGCCCGTCGTA---CGTCGAGCCGCACGT | CGTCGACGCCCTTAT

Pandoravirus inopinatum KlaHel

CTTGTTTTATGTTGT | TGCTTTTGCGTGCTG---GCAACACATGCGTCG | CAGGGCGTGGCCAGA

Pandoravirus lena

AGCGCCATTGTCCCA | TCGCCGTCGCCCATA---CAACTGGGAGCCTTG | ATGAGTCTTGCAAAA

Pandoravirus celtis

TGTGCACGCTTTGAC | GCCCTGTGTCGGATG---TTGCCCACCGTTAGC | CACGACCGTGTCGTC

Pandoravirus quercus

AAGACTCACCAAGAA | GGCTCCCAGTTGGGT---TTGCCCACCGTTAGC | CACGACCGTGTCGTC

Figure S6. Sequences around the boundaries of the 180-kbp region and its homologous region. The boundaries of the 180-kbp region and its homologous region were determined using BLASTN. "|" indicates the boundaries. Black and gray letters indicate the sequence outside and inside the regions, respectively. Fifteen nucleotides of the regions are shown, and the other parts of the regions are shown by "---".
